# Control of human gait stability through foot placement

**DOI:** 10.1098/rsif.2017.0816

**Published:** 2018-06-06

**Authors:** Sjoerd M. Bruijn, Jaap H. van Dieën

**Affiliations:** Department of Human Movement Science, Vrije Universiteit Amsterdam, Amsterdam Movement Sciences, van der Boechorststraat 9, 1081 BT Amsterdam, The Netherlands

**Keywords:** gait stability, foot placement, bipedal walking, balance

## Abstract

During human walking, the centre of mass (CoM) is outside the base of support for most of the time, which poses a challenge to stabilizing the gait pattern. Nevertheless, most of us are able to walk without substantial problems. In this review, we aim to provide an integrative overview of how humans cope with an underactuated gait pattern. A central idea that emerges from the literature is that foot placement is crucial in maintaining a stable gait pattern. In this review, we explore this idea; we first describe mechanical models and concepts that have been used to predict how foot placement can be used to control gait stability. These concepts, such as for instance the extrapolated CoM concept, the foot placement estimator concept and the capture point concept, provide explicit predictions on where to place the foot relative to the body at each step, such that gait is stabilized. Next, we describe empirical findings on foot placement during human gait in unperturbed and perturbed conditions. We conclude that humans show behaviour that is largely in accordance with the aforementioned concepts, with foot placement being actively coordinated to body CoM kinematics during the preceding step. In this section, we also address the requirements for such control in terms of the sensory information and the motor strategies that can implement such control, as well as the parts of the central nervous system that may be involved. We show that visual, vestibular and proprioceptive information contribute to estimation of the state of the CoM. Foot placement is adjusted to variations in CoM state mainly by modulation of hip abductor muscle activity during the swing phase of gait, and this process appears to be under spinal and supraspinal, including cortical, control. We conclude with a description of how control of foot placement can be impaired in humans, using ageing as a primary example and with some reference to pathology, and we address alternative strategies available to stabilize gait, which include modulation of ankle moments in the stance leg and changes in body angular momentum, such as rapid trunk tilts. Finally, for future research, we believe that especially the integration of consideration of environmental constraints on foot placement with balance control deserves attention.

## 1. Introduction

Stable gait, defined as gait that does not lead to falls [1], requires control of the position of the body centre of mass (CoM) relative to the base of support (BoS, i.e. the area within an outline of all points on the body in contact with the support surface). In gait, the BoS is formed by those parts of the feet that are in contact with the floor at any point in time. In this regard, quadrupedal animals are at a clear advantage compared to bipedal animals, yet quadrupedal and bipedal gait share common spinal neural control mechanisms in many respects, and the coordination of limb movements during walking is similar between humans [2] and quadrupeds [3]. Nevertheless, there are important differences regarding the neural control of stability. Quadrupeds can maintain stable gait in the absence of vestibular and visual feedback, relying only on somatosensory inputs and subcortical/spinal structures [4], while stable bipedal gait requires higher-order neuronal mechanisms [5].

Human bipedal gait has as a disadvantage that a large part of the total body mass is located high above a small BoS. Consequently, small deviations from a perfect body orientation result in substantial gravitational moments that accelerate the body away from this orientation and can easily move the CoM away from the BoS and lead to falls. In the sagittal plane, the body CoM moves outside of the BoS during each of the single support phases of the gait cycle. As such, during human walking, the gait pattern is not fully controllable at each moment in a step (unlike robots that walk according to a zero moment point control method), and stability must thus come from the pattern, rather than from control of the CoM within the BoS. Human gait stability can be controlled by anteroposterior (AP) placement of the foot of the swing leg relative to the body, which is also a prerequisite for forward progression [6]. To facilitate control of stability in this plane, bipedal gait may exploit the body's passive dynamics. *In silico* simulations and physical models show that stable human-like gait may exist in the absence of control [7,8], which implies that the relation between BoS and CoM may be maintained by adequate foot placement resulting from the passive dynamics [7]. However, these models cannot deal with perturbations of realistic magnitude. On the other hand, they can be stabilized through brief bursts of control, modulating either foot placement [9,10] or push-off [10,11]. In unperturbed overground gait, step length strongly covaries with gait speed [12]. When variations in step length due to fluctuations in walking speed are removed, the remaining variance is very small, suggesting that most fluctuations in sagittal plane foot placement are not used to regulate stability [12]. In addition, experimental data showed that humans do not substantially adjust sagittal plane foot placement following mechanical perturbations of gait, but do adjust centre of pressure (CoP) location (which reflects the use of ankle moments) to counteract the effect of perturbations after foot placement [13].

**Figure 1.**
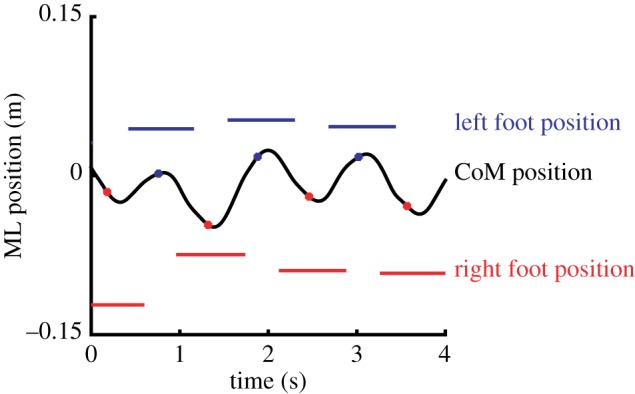
Example of mediolateral CoM motion and foot placements during normal gait. Just after midstance (coloured dots in CoM trace), the CoM starts moving to what is to become the new stance foot. Around the peak CoM velocity (not shown), the next foot placement happens, and the CoM motion starts to be redirected to the next foot. (Online version in colour.)

Stability constraints may be more dominant in the frontal plane, because the vertical projection of the CoM moves towards the lateral border of the supporting foot during each of the single-limb support phases of a gait cycle [6,14] (figure 1), which inevitably creates potential mediolateral (ML) instability (see also a recent review by Reimann *et al*. [15]). Computational models indicate that one must actively modulate the relation between the ML CoM position and the lateral border of the BoS to prevent such instabilities in bipedal human gait [16], either by controlling CoM movement through the stance leg [17] or by controlling the BoS by adjusting ML foot placement with the swing leg [17]. ML foot placement can have substantial effects on CoM acceleration, through the moment that the ground reaction force under the foot exerts on the body. Large changes in the moment arm of the ground reaction force can be achieved at relatively low actuation costs, because only the mass of the leg needs to be moved during the preceding swing phase. Consequently, as already suggested by Winter [6], foot placement appears to be the dominant mechanism for maintaining stability of bipedal gait in the frontal plane, with consistent changes in foot placement following mechanical perturbations in this plane [13,18–20]. It may be obvious that control of ML foot placement is not entirely separated from AP foot placement; yet, the literature described above (i.e. [12,13,16]) suggests that there is at least some independence. Moreover, separating these in describing them may make certain concepts more clear.

Therefore, this review focuses on the mechanisms underlying ML foot placement to control stability of bipedal gait, but we will also discuss AP foot placement at times, particularly to show why some of the developed methods do not work so well for AP foot placement. We first describe mechanical models and concepts that have been used to predict how foot placement can be used to control gait stability. Subsequently, we compare empirical findings on foot placement during human gait in unperturbed and perturbed conditions with these concepts, and review evidence on other factors that may affect foot placement. Next, we summarize the literature that attempts to answer the question of how humans achieve such a foot placement strategy, focusing on the sensory information and motor strategies involved. Finally, we conclude with a section on how disease and ageing may affect control of foot placement in humans, and what other potential strategies other than foot placement humans may use to stabilize their gait pattern.

## 2. Models for the control of foot placement

Several models to predict optimal foot placement in bipedal locomotion have been suggested. The first of these stemmed from the robotics community, where they are used to calculate where a robot should place its feet in order to prevent falling. In the following, we describe some of these models, in more or less historical order. From this description, it will become clear that the idea of foot placement to control gait stability is a recurring theme, with very similar ideas popping up in both robotics and the study of human walking.

**Figure 2.**
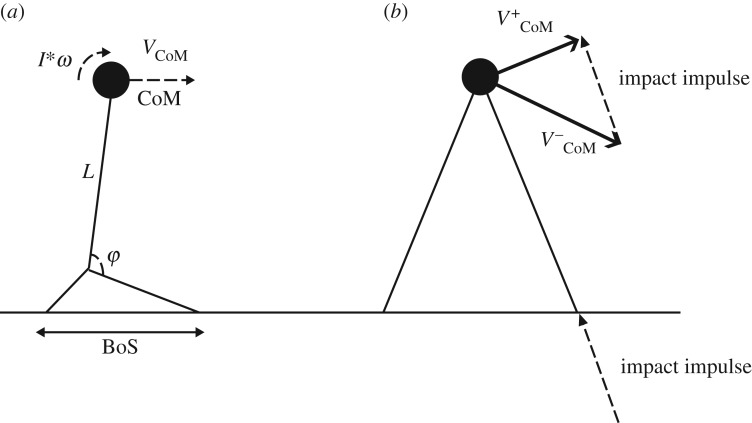
(*a*) The inverted pendulum model as used in many studies. In this model, the pendulum is assumed to rotate around the ankle joint, which is based in the foot segment, which has a certain BoS. All of the models we describe here take into account both the position (CoM) and velocity (*V*_CoM_) of the CoM. The FPE also takes into account the angular momentum around the CoM (I*ω). Furthermore, most methods further simplify this model by assuming that changes in the ankle angle (*φ*), only change the horizontal position (and velocity) of the CoM, not the vertical. Note that we draw here an AP schematic, but the same schematic holds for ML. (*b*) Finally, only the FPE method takes into account that when the BoS is shifted to regain stability, this coincides with a collision (impact impulse), which leads to the fact that the velocity after the collision 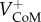 is lower than the velocity before the collision 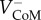, and hence, when no energy would be added to the system, the XCoM concept (and other similar concepts that do not model the impact) may predict that stability may be achieved, this may not be so.

The first to propose methods to predict foot placement as a means to stabilize human locomotion appears to have been Townsend [21], who described several strategies to stabilize bipedal gait by placing the foot relative the CoM, also taking into account CoM velocity. In these methods, the basic idea is that bipedal walking can be described by a linearized inverted pendulum model (figure 2*a*). For such a model, there is a unique combination of angle and velocity for which the pendulum will come to a standstill at the unstable fixed point. Thus, positions for the base of the pendulum (i.e. foot placement) can be formulated as a function of the CoM position and velocity, such that the pendulum will come exactly to a standstill. These models were later reinvented and implemented in actual simulations of walking machines [22,23]. Pai & Patton [24] extend this method, by also taking muscle strength into account, to calculate feasible regions of stability. However, because the method proposed by Pai & Patton [24] is expressed as a feasible region for the CoM for a given foot placement (rather than as a position to place the foot for a given CoM state), and because it is based on extensive simulations rather than a (simple) analytical expression, it has not very often been used in gait research [25,26]. The use of these methods in human movement sciences became popular via the work of Hof *et al*. [27], who coined the CoM position plus the velocity term the ‘extrapolated centre of mass' (XCoM) and used this to define margins of stability (MoS), that is distances between the edge of the BoS and the XCoM, of human walking. This MoS measure has extensively been used to quantify stability of human walking (see, for instance, [28–30]).

The models described thus far have in common that they assume a linearized inverted pendulum (i.e. one where the top of the pendulum moves in a straight line, or where inclination of the pendulum remains small) to represent gait dynamics. Wight *et al*. [31] were the first to derive these equations for a nonlinear pendulum and thereby developed a measure which they coined ‘foot placement estimator’ (FPE). A further addition of Wight *et al*. [31] was that their method included collision dynamics (figure 2*b*), by assuming a fully inelastic collision and thus conservation of angular momentum. (It should be mentioned that the work of Kajita & Tani [22] also contained a variable that could account for energy loss at heel contact.) A further advantage of the FPE is that partial derivatives of the outcome measure with respect to the assumptions can be calculated to get an idea of the errors due to violations of these assumptions. This has been done in at least two studies now, and results show that in healthy and cerebral palsy gait, violations of assumptions have little effect on the outcomes [32,33].

While these methods seem promising in that they could be used to indicate how well humans place their feet with respect to an ‘ideal’ position, there are some limitations that must be kept in mind. First of all, all of these measures are based on an (in most cases linearized) inverted pendulum model, and although such models have been shown to describe key elements of human gait [34,35], and have provided us with several key insights into it, it remains to be seen how far a description of gait dynamics using pendulum dynamics is sufficient for the purpose of finding appropriate foot placements. Secondly, these methods all calculate a position where the feet should be placed in order to come to a static equilibrium. However, in walking, it is unlikely that such a state is desirable; it would mean coming to a complete standstill, and starting again. For the AP direction, it is clear that this is not what happens when the goal is to continue walking. Research has shown that foot placement is indeed posterior to the estimated foot placement positions [32,36]. For the ML direction, one could think that it would be good to come to an equilibrium, but most research shows that foot placement is actually still lateral to estimated foot placement positions [32,36], meaning that subjects will tend to fall medially. This is understandable as it would be easier to negate (for instance by taking the next step in the direction of that fall) than a lateral fall. For the AP direction, the problem of constant CoM velocity could be addressed by, for instance, assuming constant offset control [36], or by changing the calculations of the FPE. However, these foot placement measures also assume that no energy is lost or gained during and after foot contact (with the exception of the FPE, which assumes that angular momentum remains constant, which implies a loss of energy during heelstrike). This is most likely not the case in human gait, where energy can be added by the trailing leg [37,38]. For the ML direction, if foot placement were exactly according to the estimated position, any energy added in the lateral direction would result in an outward instability (which, as stated before, cannot easily be negated by a stepping strategy). Thus, it is most likely desirable to step somewhat lateral to the estimated foot position.

Even though it suffers from the same drawbacks, as the above-mentioned methods, and has not been used in human gait research yet, we deem it worthy to also briefly mention the concept of the capture region here. This concept is similar to the XCoM, but calculates a region in which the feet can be placed to come to a stop in *N* steps, while taking into account the maximum step length that can be obtained [39].

Based on the above models of foot placement, it becomes clear that for ML control of gait stability, the feet should be placed lateral to the CoM position (see also figure 1) and even lateral to the XCoM and/or FPE. This can, of course, be achieved in at least two different ways: (i) by taking such wide steps that the feet are always placed lateral to the estimated foot placement point and (ii) by tightly regulating foot placement, so that it is just lateral to the estimated foot placement point. For the latter, both an adequate estimate of the state of the CoM with respect to the feet and sufficient ability to control the swing leg to place it at the appropriate position are needed. In the next section, we will take a look at how humans regulate their foot placement.

## 3. ML foot placement in humans

**Figure 3.**
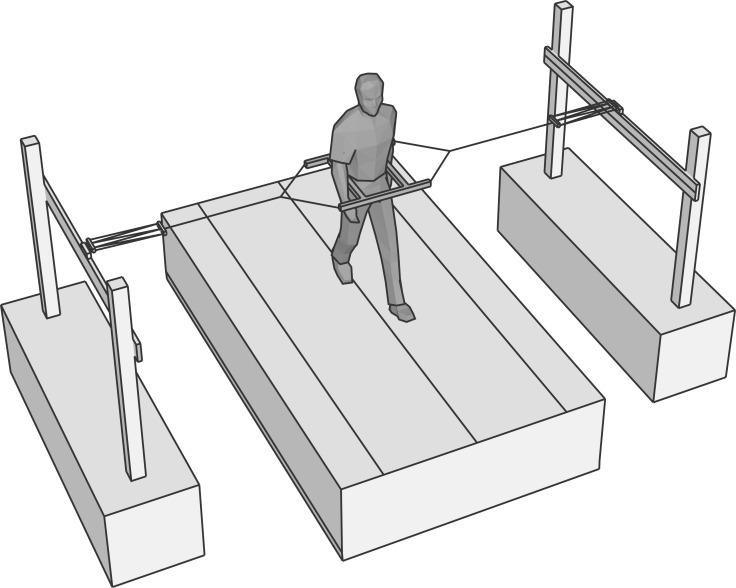
Example of a lateral stabilization set-up as used in several studies. The subject is placed in a frame, which is attached with elastic bands to the outside world. These cords are either very long or are attached to sliding rails (such as shown here), such that they do not interfere with the AP motion of the subject.

From the above, it is clear that one way to control gait stability is to coordinate ML foot placement with CoM dynamics. However, given this constraint, there is still an infinite number of positions where the foot can be placed. How do humans select step width? One idea is that they choose a step width that minimizes the energetic cost of locomotion; energetic costs of locomotion have been shown to increase when walking with a wider step width (as a consequence of the cost of redirecting the CoM velocity), but also smaller than normal step widths increase metabolic costs (as a consequence of having to swing the swing leg around the stance leg) [40,41]. The idea that redirection of the CoM velocity involves substantial metabolic costs in walking is further strengthened by several studies that show that lateral stabilization by means of elastic bands (figure 3) leads to smaller step widths [17,42–44] and may reduce the metabolic cost of locomotion [42–45], although the latter has not been found consistently, and we recently failed to find this effect ourselves (see https://osf.io/gkphs/). Thus, humans appear to choose an average step width that minimizes, or at least limits, energy costs.

Still, average step width may not tell the whole story. For instance, Wezenberg *et al*. [46] found that enforcing subjects to walk with their average step width increased metabolic cost and variability of the CoP of the ground reaction force. This suggests that a control strategy based on a fixed step width is not optimal and requires additional control effort through the stance leg. Thus, it appears that humans do not simply choose a certain step width, but actively coordinate foot placement with respect to CoM movement each step.

### 3.1. Coordination of foot placement and kinematic state

The previous section illustrated that humans appear to modulate the relation between the ML CoM position and the lateral border of the BoS to control stability [16], which can be done either by controlling CoM movement through the stance leg [17] or by controlling the BoS by adjusting ML foot placement with the swing leg [17,47–50]. Note that the ML boundary of the BoS can also be adjusted through toeing out, a strategy that may be used when foot placement is constrained [51]. Hypotheses on control of foot placement can be tested using, for instance, the uncontrolled manifold concept [52], which assesses how far joint-level variability covaries with respect to the (supposedly) controlled variable. Using this approach, Verrel *et al*. [53] found that there is a strong covariation of joint-level variability such that the foot placement with respect to the CoM position is stabilized. Moreover, when walking on a narrow beam, variability in joint angles increased, while, as expected, variability in step width actually decreased [54]. This suggests a tighter control of these joint angles to achieve the more precise foot placements.

While covariance of joint kinematics to aid reduction in foot placement variability suggests that foot placement with respect to the CoM state is actively controlled, it does not tell us *how* it is controlled. As the foot cannot be simply placed at any given point at any given time, the information of where the foot should be placed should, in some way, be available well before the foot is placed. Using the correlation between the trunk CoM kinematics and foot placement, Hurt *et al*. [47] showed that CoM state (position and acceleration) at midstance is predictive of where the following foot placement will be. On the group level (i.e. over both between and within subject variance), the trunk state predicted 53% of variance in foot placement. Note that trunk CoM state was used here rather than whole-body CoM. This choice was motivated by the fact that the trunk represents a large proportion of body mass and that control of the trunk mass is key in maintaining stable gait [55]. In later work, Wang & Srinivasan [48] used a similar approach, but based their analysis on individual subject data, thereby showing that as much as 80% of the variance in deviations of foot placement from the average could be explained by deviations in pelvis position and speed from average at midstance. In gait, pelvis position is a reasonable proxy for CoM position [56]. Interestingly, these data-driven models for foot placement during gait agree to a large extent with the earlier described theoretical models in that the CoM kinematic state and not just the CoM position are used to select foot placement and add information on *when* in the gait cycle foot placement is chosen.

The association between CoM state and foot placement described above has been interpreted as reflective of active control, but could also result from passive dynamic coupling of movements of the leg to the movements of the upper body. With increasing prescribed step width, the gain of the coupling between CoM state and foot placement decreased [49], suggesting a form of active control that is relaxed under less demanding conditions. Further support for active regulation of stability through foot placement is provided by studies on walking with lateral stabilization. This manipulation decreases not only lateral displacement of the CoM, and step width, but also leads to a decrease in step width variability [44,45,57], even if trunk kinematics are constrained without any coupling to the external world [17]. Moreover, studies using mechanical perturbations of gait showed that adjustments of foot placement were correlated with the induced change in CoM velocity [13,19,20,58] and that these adjustments were actively generated [20,58]. Lastly, an increased ability of the CoM state to predict foot placement with increasing walking speed [50] suggests that such control increases with walking speed (at least for speeds up to 1.2 m s^−1^; for higher walking speeds, step width seems to increase again [59], although it is not clear whether this also directly indicates a decrease in control). All in all, this body of evidence clearly supports the idea that ML foot placement is regulated based on the CoM state in the preceding swing phase.

### 3.2. Sensory information for estimation of centre of mass state

The finding that foot placement is coordinated in relation to the kinematic state of the CoM in the preceding swing phase raises the question how the brain estimates the CoM state. It might use sensory information from three modalities: the proprioceptive, visual and vestibular systems. Studies using visual perturbations of gait have shown compensatory trunk movements [60] and changes in foot placement [61] and, with continuous unpredictable visual perturbations, variability of both trunk movement and foot placement increased [62]. Vestibular stimulation [63–67] and proprioceptive stimulation through the vibration of trunk or neck muscles [68] lead to ample deviations of heading. These results may indicate a role of vestibular and proprioceptive feedback in controlling the heading rather than in stability control. However, the trajectory deviations may, at least in part, result from perturbations of stability, leading to compensatory sideward stepping [63] and concomitant external rotation of the foot [51]. This is supported by work of our group, showing that multisine vestibular stimulation increases variability of ML trunk kinematics and decreases ML gait stability [69], and by studies showing that muscle vibration, a means to manipulate proprioceptive afference from muscle spindles, during the stance phase of gait caused compensatory trunk movements [70] and changes in ML foot placement [71]. The literature thus suggests that each of the three sensory modalities considered contributes to estimation of the CoM state and adjustment of ML foot placement to control stability, but this raises the question how multisensory information is integrated.

The visual and vestibular systems provide information about the orientation and motion of the head in space, which must be combined with information about the motion of the head relative to the trunk (proprioception) to provide an estimate of the CoM state (i.e. position, velocity and higher-order derivatives) [72]. Furthermore, each sensory modality has its specific latency and filtering characteristics. Across many tasks and modalities, multisensory information is assumed to be combined as a weighted average, with weights based on the basis of the relative reliability of the separate sources, which, for steady-state behaviour, can be defined as the inverse of the variance of the source [73,74]. The relative reliability of the various sensory information sources can be studied in standing with relative ease [75], and methods have been developed to estimate their contributions to stability control in static situations such as upright sitting and standing [76,77]. However, it is likely that sensory contributions are different between standing and walking. First, the reliability of sensory signals is different during walking and might vary over the gait cycle, given variations in amplitude and frequency of inputs during different phases of gait. Second, in addition to direct sensory information, state estimation is likely influenced by prior information, for instance based on an efference copy of the motor command [78,79], which will differ between standing and walking, and will again be time-varying in the latter context. Empirical data indeed support that sensory weighting is different in walking than in standing. For example, vibration of the leg muscles had much more pronounced effects in standing than in walking [68], while effects of visual perturbations were larger in walking than in standing [60]. Moreover, the importance of visual perturbations in walking appears to be directionally specific with larger effects of ML perturbations than those of AP perturbations [60,61].

Empirical data indicate that in control of static postures, the central nervous system adapts by reweighting the sensory inputs that contribute to stability [76,80–83]. Similarly, weighting of multisensory information for gait stability may vary over time when conditions change, making sensory inputs less reliable. Such reweighting of information may actually be more important in walking than in standing, as the process of locomotion itself may induce variation in sensory environments, such as when walking from a well-lit into a dark room, or when stepping from a solid onto a compliant surface. Although some evidence suggests that the role of proprioception is much reduced in gait compared to stance [68], it has been observed that after sufficient habituation time, blindfolded individuals display a near-normal gait pattern [84], implying that stability can be maintained by relying on remaining sensory inputs. It thus seems reasonable to assume that the brain exploits the redundancy provided by the three sensory modalities by reweighting inputs when one becomes less reliable. However, sensory reweighting in gait has, to our knowledge, not been studied.

As suggested above, reliability of sensory information may vary over the gait cycle, and, hence, weighting of sensory information may also vary over these shorter time scales. Vestibular stimulation in different phases of the gait cycle caused systematic variation in responses of lower limb muscles [85,86] and in the magnitude and timing of deviations in ML foot placement [64,87]. In addition, H-reflex amplitudes in human calf muscles are phase-dependent, but it is not clear whether this reflects modulation of motor unit excitability or modulation of proprioceptive feedback gain [88]. These studies suggest that the effects of sensory inputs during gait are phase-dependent, but it is not clear whether and how these modulations are relevant to foot placement.

In conclusion, it is evident that multisensory information, based on vestibular, visual and proprioceptive inputs, is used to maintain stability of gait. The weighting of the sensory inputs likely depends on environmental conditions and the related change in reliability of the information provided by any single input, and is likely to vary across the gait cycle, but the dynamics of these weighting processes are largely unexplored.

### 3.3. Actuation of foot placement

As outlined in paragraph §3.1, foot placement can already be predicted at midstance. How then, does the musculoskeletal system make sure that the foot gets to the correct position half a step later? Recent research has shown that this is, at least in part, controlled by an activity of the swing-leg gluteus medius muscle. In both unperturbed walking and walking with ML mechanical perturbations, swing-phase gluteus medius activity was associated with more lateral foot placement and predicted by the ML distance between the CoM and the contralateral stance foot [58]. The gluteus medius activity after ML perturbations were shown to occur in bursts at latencies of 100 and 170 ms, respectively, indicating automatic involuntary muscle activity, and a later burst at a latency of more than 270 ms, most likely voluntary in nature. These responses were phase-dependent, showing facilitation after perturbation in the swing phase and no response in the stance phase, in contrast to the normal walking (background) activity [20]. The hip joint moment required to accelerate the swing leg and the associated muscle activity are relatively low, and an experimentally induced reduction of strength of the hip by as much as 26% though a partial nerve block had no effect on frontal plane trunk and leg kinematics [89], illustrating that this control strategy is quite robust.

### 3.4. Neural control of foot placement to control gait stability

Although we have discussed how human gait stability can be controlled through foot placement, what sensory information is required to do so, and by which muscles such foot placement strategies are executed, we have not yet discussed which parts of the central nervous system might be involved in this control. While reflexes may play a role in accurate foot placement [20], studies relating white matter lesions and brain atrophy to falls suggest that higher centres in the central nervous system also play an important role [90–92]). In a review of the literature, Zheng *et al*. [90] concluded that white matter lesions in the frontal lobe and periventricular regions have strong relationships with balance and gait measures, suggesting that these regions could be involved in selecting and guiding foot placement.

Some studies have more directly assessed the relationship between brain metrics and measures related to foot placement strategies. For instance, decreased trunk stability (which could be seen as a proxy of CoM control) during dual tasking has been suggested to coincide with greater brain atrophy [93]. Moreover, using positron emission tomography, Shimada *et al*. [94] showed differences in gait-related (de)activations in the primary sensory motor area, middle and superior temporal gyrus, and hippocampus between groups with low and high step-length variability, suggesting a role for these areas in control of foot placement. Additionally, using diffusion tensor imaging (a method to assess white matter integrity), Bruijn *et al.* [95] showed that higher quality of white matter in the left corticospinal tract and left anterior thalamic radiation coincided with higher MoS (i.e. the distance between the XCoM and the edge of the BoS at foot placement), suggesting an important role for these tracts in the control of foot placement.

More recent studies have employed electroencephalography (EEG) to understand the role of higher centres of the central nervous system in maintaining gait stability, which has the advantage that it can be used during actual gait. Using this approach, Sipp *et al*. [96] showed that β activity in left and right sensorimotor areas decreased (a sign of increased motor control) during balance beam walking when compared with normal walking. Taking the opposite approach (i.e. by stabilizing subjects), Bruijn *et al*. [97] showed that β activity in the left premotor cortex was lower during normal than stabilized walking, which further supports the role of these brain areas in control of gait stability. Moreover, using effective connectivity measures, a recent study confirmed that at least part of this activity is driving the muscles [98]. All in all, it seems that apart from control from a spinal [20] level, higher centres of the central nervous system are actively involved in controlling foot placement to maintain stability.

## 4. Discussion

While for most of us walking on two legs is not a great feat, at both ends of the age spectrum, it is obvious that our bipedal gait is far from trivial. Especially in elderly people, falls may have devastating effects. However, knowledge on how we are able to walk on two legs is limited, which may also hamper our ability to improve gait stability in populations in need. In the current review, we synthesized our current understanding of how appropriate foot placement can contribute to bipedal locomotion without falling. We have shown that ML foot placement is critical for gait stability, that such ML foot placement can be predicted from mechanical models, and can be identified from human gait data. Furthermore, we have identified sensory and motor contributions that are needed for the successful execution of these foot placement strategies. In this section, we will discuss how the use of these strategies can become impaired and how this affects gait stability. We will mainly focus on the effects of ageing, with some reference to pathological gait. Subsequently, we address alternative strategies to foot placement to control gait stability. Finally, we will reiterate the gaps in our current understanding, and, as such, indicate directions for future research.

### 4.1. Effects of ageing on control of gait stability through foot placement

As outlined in §3, control of gait stability requires adequate coordination between the CoM state and foot placement, which in turn requires adequate sensing of both CoM state and foot location, and adequate muscle activity to direct the swing foot to the correct location. Thus, it should come as no surprise that any condition that impairs sensory or muscle function may impair gait stability due to an impaired ability to control foot placement, as becomes apparent with ageing and pathology.

Older adults generally walk with wider steps than young adults [99,100]. Similarly, in pathology that affects sensory and/or motor function, larger step widths are often observed [18,30,101–103]. This increased step width may be adaptive, because, for example, older adults have been reported to show larger and faster ML CoM movements than young adults [104]. Such an interpretation is supported by the fact that a narrower step width in older adults is associated with higher fall risk [105] and by the fact that healthy adults also increase step width when balance is challenged by external perturbations [106]. However, in older adults, the increase in step width was found not to be sufficient to prevent a smaller MoS than in young adults [104]. Moreover, given inconsistencies in the literature [95], it seems that not all adults adapt step width, and it has been indicated that the lack of such adaptations is associated with white matter degeneration in pathways involved in control of gait stability [95]. It should be noted that, in contrast with the interpretation of increased step width as being adaptive, wider steps are likely to contribute to the increase of ML CoM sway [49,107].

Hurt *et al*. [47] found weaker correlations between the trunk kinematic state at mid-swing and subsequent foot placement in older than young adults. While this may suggest a less accurate coordination between foot placement and CoM kinematics, the analysis was performed at a group level, and may hence be affected by differences in between-subject variance between old and young subjects. In line with a loss of coordination with ageing, Arvin *et al.* [17,104] found not only a more variable step width, but also a more variable MoS in older adults. In stroke patients, impaired precision in performing a hip abduction tracking task was associated with wider steps of the paretic leg [108]. This suggests that precision of control over the swing leg may limit coordination between CoM movement and foot placement. It is as yet unclear if and how impairments in motor, sensory and/or neural function cause such impaired coordination.

### 4.2. Alternatives for foot placement

Up to now, we have considered the case where foot placement is free, and entirely guided by the need to control stability. As discussed, in these cases, CoM state predicts foot placement to a large extent. However, there may be situations in which foot placement is constrained, and, thus, it is not possible to control gait stability by foot placement. When a selected foot placement location is blocked, new foot placement locations are selected to minimally deviate from the planned location [109,110], which can be understood in terms of minimizing the effects of the alternative foot placement location on gait stability [110,111] and underscores the importance of selection of foot placement locations. In addition, Matthis & Fajen [112,113] have shown that in these cases, CoM kinematics are also adjusted, such that they match foot placement. In particular, they showed that when subjects can see two or more steps ahead, their CoM kinematics remain (more or less) ballistic (i.e. without sacrificing an energetically optimal strategy).

When foot placement is constrained, it can obviously not be used as main mode of control. The findings of Matthis & Fajen [112,113] thus suggest control of CoM kinematics in relation to the planned foot placement. In line with this, subjects walking over a narrow path, which constrained foot placement to a location medial from normal, were shown to reduce their CoM amplitude and velocity [17], although control of foot placement with low variability in this situation actually coincides with more variability in joint angles [54]. Mechanically, there are two alternative strategies that can be used to control the CoM [114]: (i) moving the CoP of the ground reaction force by generating appropriate moments around lower extremity joints and (ii) changing the direction of the ground reaction force by changing the angular momentum of segments around the CoM.

Hof *et al*. [18] have provided evidence for the use of the first mechanism; they showed that when the CoP was close to the BoS at the beginning of a step, it tended to move outwards during the stance phase. They suggested that the inability to apply such corrections was a factor in the use of a large MoS in amputees compared to healthy controls. Later studies showed that CoP shifts are also used after perturbations [13,19]. In a recent study, Kim & Collins [115] showed that in amputees, the effort associated with balance (e.g. energetic cost) could be reduced by appropriate control (inversion/eversion torque) of a robotic prosthesis, further highlighting the importance of stance leg control. It should be noted here that in walking, changing the magnitude of the ground reaction force at a constant CoP position can be used to control the CoM in a similar way to displacement of the CoP. This allows for control of ML stability by modulation of the push-off force [10], given the lateral offset of the trailing foot with respect to the body. Using a powered ankle exoskeleton, Kim & Collins [116] showed that appropriate modulation of the push-off force based on the CoM state reduced the effort associated with maintaining stability during walking, hereby further highlighting the potential role of the ML component of push-off to stabilizing human gait.

For the second mechanism, Neptune & McGowan [117] studied which muscles contribute to frontal plane stability. By calculating the contribution of these muscles to angular momentum at each moment in the gait cycle, they showed important contributions of the plantar flexors during push-off, as well as the hip abductors during single stance. Moreover, Fu & Kuo [118] recently reported that ML perturbations applied towards the trailing leg in early stance (i.e. when the leading leg is an obstacle for adjusting foot placement by means of a cross-over step), trunk rotation in the direction of the perturbation was used to counteract the effect on CoM kinematics. This is agreement with a strategy that is aimed at generating desirable angular momentum to change CoM acceleration.

All in all, the literature indicates that besides foot placement as the dominant mechanisms for control of ML stability in gait, other mechanisms are used. It is, to a large extent, unclear how these strategies interact, and how this may differ depending on the phase of the gait cycle, environmental context, impairments due to ageing or pathology.

### 4.3. Directions for future research

We are beginning to understand how humans control their gait stability through foot placement, yet many open questions remain.

In §§2 and 3.1, we discussed several models that can be used to predict where humans place their feet. Some of these models are currently being used to derive stability metrics, yet it is not clear if and how they correlate to manifestations of instability, i.e. to falls. Moreover, while the empirical models of §3.1 explain a large proportion of variance in observed foot placement, it is obvious that they only capture stabilization through foot placement, whereas other strategies (§4.2) are also of importance; it remains to be seen whether these strategies can be incorporated in a more comprehensive model of the control of gait stability. Lastly, the described models for foot placement estimation do not take into account that AP and ML foot placement may interact; for instance, when walking with longer steps, given finite leg length, the step width that can be obtained is smaller. This might imply that faster walking would lead to fewer possibilities to correct ML instabilities or, conversely, that people who walk with very wide steps may have problems attaining reasonable walking speeds. But this would also suggest that the assumption of separate control of AP and ML stability made here, and in the literature reviewed, is not appropriate. It is unknown to what extent such interactions are relevant in daily-life gait, and if and how these are controlled.

In §3.2, we described the sensory information that could be used to estimate the CoM state during walking. In this section, it became obvious that for standing phenomena like sensory reweighting are well studied, but for walking, open questions on the relative importance of various sensory modalities, on their phase-dependency and on reweighting remain.

We are only beginning to understand how different levels of the central nervous system are involved in controlling foot placement to maintain gait stability. Important advances in this field will most likely come from the *combination* of metrics obtained from the central nervous system with gait stability metrics, and/or conditions in which the (need to control) gait stability is altered. For instance, it would be interesting to investigate if, and how much, the burst of gluteus medius activity during the swing phase [58] is controlled from a cortical level. This could be studied by means of directed functional connectivity between EEG and EMG signals [98], in combination with situations in which the need to control foot placement is more (or less) important. We stress that in studying the role of the central nervous system to control foot placement, not only the role of the higher levels should be evaluated, as lower levels, such as for instance the spinal cord and brainstem, may also play an important role.

In ageing (§4.1), there appears to be an impaired coordination between CoM movement and foot placement. It is as yet unclear if, and how, impairments in motor, sensory and neural function cause such impaired coordination.

Lastly, while we discussed that there are cases in which foot placement cannot be used to control gait stability (for instance, when walking on constrained footholds, see §4.3), and the strategies that can be used in such cases, it is unclear in how far in daily-life gait stability is controlled by foot placement strategies, or in how far step locations are visually selected first, after which the alternative strategies are used. Moreover, how these strategies are integrated in the control of gait stability remains unknown. For instance, how is switching between strategies achieved and does last-moment switching from, for example, a foot placement-based strategy to a foothold-based strategy when avoiding a puddle, impose specific challenges? Novel work by Matthis *et al*. [119] in which eye tracking and motion capture are performed in real-life challenging environments has begun to unravel such issues.

## Data accessibility

This article has no additional data.
